# Supplementary material for: Wildflower phenological escape differs by continent and spring temperature
Source: Nat Commun. 2022 Nov 22;13:7157. doi: 10.1038/s41467-022-34936-9 (PMC9684453; doi:10.1038/s41467-022-34936-9)
Supplement: Supplementary file 1 — Supplementary Information [file 41467_2022_34936_MOESM1_ESM.pdf]

Wildflower phenological escape differs by continent and spring temperature

Authors: Benjamin R. Lee, Tara K. Miller, Christoph Rosche, Yong Yang, J. Mason Heberling, Sara E. Kuebbing, and Richard B. Primack

Supplementary Methods

Model design and spatial autocorrelation

Summary of INLA spatial autocorrelation analysis

There were no significant differences (and high similarity in mean values) among the estimated intercepts or phenological sensitivities to spring temperature and elevation when comparing the posterior estimates from the analogous JAGS and INLA-SPDE models (Table S4). This suggests that the high fidelity between the two approaches found by other studies apply to our phenological models as well.

In contrast, incorporating spatial autocorrelation into the model structure changed model fit and performance as well as posterior estimates of parameter values, sometimes in statistically significant ways (Tables S3-S4). The model versions that included spatial autocorrelation had elevated predicted vs. observed r^2 values and significantly lower DIC values compared to those that did not for all combinations of plant group and continent (Table S3). Specifically, models that included spring temperature and elevation as fixed effects, species as a random effect, and the spatial autocorrelation term were the best-performing or among the best-performing model structures for five of the six plant group-continent combinations (the only exception being tree LOD in Asia, which was better predicted by either including winter temperature or annual precipitation instead of elevation).

Including spatial autocorrelation had a particularly strong effect on model fit and performance in Asia and Europe (Table S3), where predicted vs. observed r^2 values increased by 69-86% and 50-65%, respectively. North American models, which had the best-performing

models prior to the addition of spatial autocorrelation terms, only experienced increases in predicted vs. observed r^2 values of 3-6%. In contrast to these large differences in how model performance was affected, the only parameter values that significantly changed with the addition of spatial autocorrelation terms were in the North American models (Table S4). North American phenological sensitivity to spring temperature was significantly less negative with the addition of spatial autocorrelation terms for both wildflower FFD and canopy tree LOD models. Importantly, the difference in phenological sensitivity between trees and wildflowers that was statistically significant in the original model structure disappeared – there was no significant difference in sensitivity to spring temperature when spatial autocorrelation was included in the model. This was the only continent where the relationship in spring temperature sensitivity between wildflowers and canopy trees changed in response to adding spatial autocorrelation into the model. Model intercepts and sensitivities to elevation did not significantly change for any plant group-continent combination when spatial autocorrelation was added to the model structure (Table S4).

Justification for exclusion of spatial autocorrelation

The addition of the spatial autocorrelation term had qualitatively different effects depending on the continent. This is most noticeable for the Asian models, where the posterior-predicted mean effects of the spatial autocorrelation term ranged from -37.2 to 50.1 days for trees and -38.8 to 36.0 days for wildflowers (Fig. S2), meaning that the spatial relationship between data points altered predicted phenology by more than a month in either direction even after spring temperature and elevation had been accounted for in the model structure. This range was narrower in both Europe (Fig. S3; -10.8 to 6.0 days for trees and -24.6 to 33.1 days for

wildflowers) and eastern North America (Fig. S4, -18.8 to 19.3 days for trees and -10.3 to 9.5 days for wildflowers), suggesting that spatial relationships (which could include relationships with climate or geographic drivers that covary with the spatial relationships) are more influential on the predictive accuracy of spring phenology models in Asia compared to on the other two continents.

Potentially even more apparent when comparing Figures S2-S4 is the difference in the smooth clines in North America and the patchy, hot-spot patterns in Europe and Asia. The patterns in North America appear to follow latitudinal trends, potentially reflecting photoperiodic effects that have been previously found to strongly affect spring phenology of deciduous tree species[1]. Europe is somewhat like North America in that there are relatively smooth gradients that appear to be somewhat linked to geographic features (for example, strong spatial autocorrelative effects appear for wildflower species along major mountain ranges). However, different ranges have different effects on phenology with delayed flowering in the Pyrenees and earlier flowering in the Alps. On one hand, this makes some sense because our models already account for elevational effects, but on the other hand, it seems to contradict other potential drivers associated with mountains such as the timing of snowmelt. It is also interesting to note that our results qualitatively differ from a recent European study also using herbarium collections to model spring phenology shifts[2], which found early-shifts associated with both mountain ranges when elevation, year, spring and winter temperature, and spring precipitation had all been accounted for as phenological drivers.

Sharply contrasting the North American and European results, however, spatial autocorrelation in East Asia appears extremely heterogeneous, with regions of extremely delayed phenology immediately next to regions with strong early shifts (Fig. S2). We could think of no

reasonable explanations to explain the observed patterns of spatial autocorrelative effects (for example, some adjacent areas differed by a total of about 2 months in predicted effects) and therefore posit that they are the result of nuances in where and how the herbarium specimens were collected. In support of this hypothesis, Asian spatial autocorrelation had overall much wider standard deviation (Fig. S2b, d) compared to both Europe and North America, suggesting a greater degree of uncertainty in the spatial relationships inherent to the collections. This appears to be, in part, due to the concentration of collections in certain areas (i.e., the areas with much lower standard deviations). That is, areas with strong spatial autocorrelational mean effects are typically also the areas with the lowest standard deviation, suggesting that collections are concentrated in these areas. This could therefore also suggest that some regions were simply collected earlier on average than others as a function of logistical constraints (e.g., it is easier to collect specimens closer to urban areas as opposed to rural areas) rather than through accurate reflection of ecological processes.

Due to the inconsistencies in how spatial autocorrelation affected predicted phenology across the three continents, we decided not to include this term in the models presented in the main body of the manuscript. Still, we want to highlight the importance in how these effects can influence model performance both in terms of predicted effects (e.g., spring temperature sensitivity changed for North American plant species depending on if spatial autocorrelation was included in the model or not) and in terms of predictive power (European and Asian models experienced substantial increases in predicted vs. observed r^2 values when spatial autocorrelation was added to the models). We would also like to note that because the nuances and discrepancies that arose with the Asian phenology models are likely related to concentrated observations and

uneven sampling distributions, further collections and digitization efforts should resolve these biases in the near future.

Supplementary Tables

Table S1: Number of individual specimens of each species used in this study. Total number of specimens across all continents N = 5,522.

Region	Lifeform	Species	#Specimens
Eastern Asia	Trees n = 899	*Acer pictum subsp. Mono* (Maxim.)	37
		Acer truncatum (Bunge)	94
		Betula platyphylla (Sukaczev)	113
		Fraxinus chinensis (Roxb.)	99
		Juglans mandshurica (Maxim.)	53
		Populus davidiana (Dode)	125
		Quercus aliena (Blume)	44
		Quercus mongolica (Fisch.)	66
		Quercus variabilis (Blume)	186
		Ulmus pumila (L.)	82
	Wildflowers n = 1,418	*Androsace umbellata* (Lour.)	486
		Astragalus scaberrimus (Bunge)	203
		Gueldenstaedtia verna (Georgi)	185
		Leibnitzia anandria (L.)	206
		Pulsatilla chinensis (Bunge)	177
		Viola prionantha (Bunge)	161
Europe	Trees n = 532	*Acer platanoides* (L.)	96
		Acer pseudoplatanus (L.)	69
		Carpinus betulus (L.)	62
		Fagus sylvatica (L.)	184
		Quercus petraea (Matt.)	54
		Quercus robur (L.)	67
	Wildflowers n = 618	*Allium ursinum* (L.)	79
		Anemone nemorosa (L.)	130
		Anemone ranunculoides (L.)	52
		Corydalis cava (L.)	124
		Corydalis solida (L.)	50
		Hepatica nobilis (L.)	183
Eastern North America	Trees n = 995	*Acer rubrum* (L.)	308
		Acer saccharum (Marshall)	138
		Carya glabra (Miller)	111
		Fagus grandifolia (Ehrh.)	161
		Quercus alba (L.)	158
		Quercus rubra (L.)	119
	Wildflowers n = 1,060	*Anemone quinquefolia* (L.)	188
		Dicentra canadensis (L.)	143
		Dicentra cucullaria (L.)	169
		Erythronium americanum (Ker-Gawl.)	191
		Hepatica americana (DC.)	148
		Sanguinaria canadensis (L.)	221

Table S2: Means and standard deviations of day of year of observed phenology and estimated spring temperature of each observation, grouped by continent and lifeform (trees or wildflowers).

Continent	Group	Observed Day of Year		March-April Temperature (°C)	
		Mean	S.d.	Mean	S.d.
Asia	Trees	115.72	24.83	9.12	4.57
	Wildflowers	116.77	24.44	8.48	4.32
Europe	Trees	132.24	15.12	5.54	2.44
	Wildflowers	115.92	20.75	5.01	2.80
N. America	Trees	116.87	22.57	8.84	5.42
	Widlflowers	110.55	19.00	7.19	4.52

Table S3: Model comparison statistics for the different model versions evaluated as part of this study. Values in bold italics indicate the model(s) with the best deviance information criterion (DIC) value, assessed as being at least two less than all other models for the combination of continent and plant lifeform (wildflower or tree).

Region	Model	Wildflower FFD		Canopy Tree LOD	
		r^2	DIC	r^2	DIC
Asia	Spring T	0.388	12407.66	0.437	7832.79
	SpringT + S.A.	***0.740***	***11881.22***	0.738	7556.18
	Spring T + Elevation	0.398	12385.98	0.437	7833.75
	Spring T + S.A. + Elevation	***0.739***	***11881.01***	0.738	7557.06
	Spring T + S.A. + Winter T	0.739	11885.25	***0.740***	***7551.11***
	Spring T + S.A. + Annual Prec.	0.739	11886.79	***0.739***	***7551.04***
	Spring T + S.A. + Spring Prec.	***0.741***	***11882.06***	0.737	7557.83
Europe	Spring T	0.356	5246.99	0.236	4270.80
	SpringT + S.A.	0.678	5012.63	0.387	4248.59
	Spring T + Elevation	0.407	5198.44	0.252	4261.37
	Spring T + S.A. + Elevation	***0.672***	***5005.54***	***0.380***	***4237.75***
	Spring T + S.A. + Winter T	0.678	5013.31	0.370	4248.04
	Spring T + S.A. + Annual Prec.	0.679	5013.94	0.388	4248.09
	Spring T + S.A. + Spring Prec.	0.678	5014.48	0.382	4249.59
North America	Spring T	0.705	7968.63	0.754	7646.52
	SpringT + S.A.	0.725	7939.67	0.814	7498.66
	Spring T + Elevation	0.705	7969.54	0.761	7621.36
	Spring T + S.A. + Elevation	***0.726***	***7927.93***	***0.809***	***7483.74***
	Spring T + S.A. + Winter T	0.733	7943.83	0.814	7500.58
	Spring T + S.A. + Annual Prec.	0.731	7941.40	0.814	7500.18
	Spring T + S.A. + Spring Prec.	0.725	7939.06	0.814	7499.84

Table S4: Posterior estimated mean and 95% Bayesian credible intervals of model slopes and intercepts for models that include average spring temperature and elevation as fixed effects. Posterior estimates are presented for models both with and without spatial autocorrelation terms.

			**Sp. Temp. + Elev. (JAGS)**			**Sp. Temp. + Elev. (INLA)**			**Sp. Temp., Elev., + S.A.**		
			**2.5% CI**	**Mean**	**97.5% CI**	**2.5% CI**	**Mean**	**97.5% CI**	**2.5% CI**	**Mean**	**97.5% CI**
Asia	FFD	β_1 (SpT)	-3.364	-3.118	-2.861	-3.382	-3.131	-2.879	-3.417	-3.053	-2.686
		Intercept	96.78	120.46	144.11	111.33	134.90	158.46	112.31	136.09	159.85
		β_2 (Elev)	2.335	3.794	5.242	2.239	3.733	5.227	0.406	2.940	5.482
	LOD	β_1 (SpT)	-2.971	-2.621	-2.261	-3.000	-2.647	-2.293	-2.834	-2.354	-1.865
		Intercept	110.57	129.37	148.13	119.28	138.30	157.31	116.45	135.98	155.49
		β_2 (Elev)	-2.544	-0.840	0.882	-2.668	-0.894	0.878	-2.530	0.335	3.263
Europe	FFD	β_1 (SpT)	-3.476	-3.021	-2.565	-3.510	-3.044	-2.578	-3.833	-3.245	-2.656
		Intercept	85.64	108.91	132.54	99.49	123.11	146.70	103.35	127.29	151.21
		β_2 (Elev)	10.722	14.768	18.678	10.694	14.682	18.666	6.940	12.538	18.103
	LOD	β_1 (SpT)	-3.268	-2.792	-2.298	-3.310	-2.815	-2.321	-3.440	-2.853	-2.259
		Intercept	101.39	125.22	149.10	116.32	139.94	163.55	115.51	139.31	163.08
		β_2 (Elev)	3.094	7.098	11.162	2.997	7.093	11.187	4.293	9.649	15.297
North America	FFD	β_1 (SpT)	-3.276	-3.141	-3.004	-3.279	-3.141	-3.002	-2.793	-2.403	-2.009
		Intercept	91.11	113.95	136.98	104.66	128.16	151.63	91.06	119.70	147.39
		β_2 (Elev)	-1.394	1.779	4.951	-1.467	1.715	4.894	2.854	7.189	11.565
	LOD	β_1 (SpT)	-3.756	-3.617	-3.485	-3.752	-3.619	-3.485	-2.865	-2.422	-1.977
		Intercept	104.03	127.07	149.88	117.90	141.40	164.89	105.11	131.68	157.58
		β_2 (Elev)	6.863	10.835	15.037	6.733	10.765	14.793	8.502	14.257	20.009

Table S5: Posterior estimated species-level random effects (means and 95% Bayesian credible intervals) for East Asian tree and wildflower species. Estimates are provided for the parameters derived from the JAGS model described in the main methods section.

	**Posterior estimates for JAGS model**		
Tree Species	2.5 BCI	Mean	97.5 BCI
Acer pictum ssp. *mono*	-4.02	16.032	34.763
Acer truncatum	-7.489	11.93	31.63
Betula platyphylla	-3.439	16.033	34.84
Fraxinus chinensis	-0.761	18.535	37.14
Juglans mandshurica	3.872	23.562	42.211
Populus davidiana	-20.846	-1.648	17.295
Quercus aliena	-1.879	17.659	37.179
Quercus mongolica	4.582	24.411	43.4
Quercus variabilis	-8.225	11.018	30.302
Ulmus pumila	-33.017	-12.825	6.226
Wildflower Species			
Androsace umbellata	-2.874	20.763	44.233
Astragalus scaberrimus	1.773	25.941	49.428
Gueldenstaedtia verna	-2.861	21.045	43.999
Leibnitzia anandria	-0.886	22.688	46.489
Pulsatilla chinensis	-6.085	17.687	41.292
Viola prionantha	-11.351	12.456	35.209

Table S6: Posterior estimated species-level random effects (means and 95% Bayesian credible intervals) for European tree and wildflower species. Estimates are provided for the parameters derived from the JAGS model described in the main methods section.

	**Posterior estimates for JAGS model**		
Tree Species	2.5 BCI	Mean	97.5 BCI
Acer platanoides	-10.918	12.977	36.649
Acer pseudoplatanus	1.272	25.373	49.333
Carpinus betulus	-9.569	14.034	38.254
Fagus sylvatica	-2.973	20.898	44.675
Quercus petraea	4.917	28.551	52.648
Quercus robur	1.771	25.543	49.512
Wildflower Species			
Allium ursinum	16.907	40.499	64.238
Anemone nemorosa	-8.603	15.288	38.582
Anemone ranunculoides	-5.891	17.459	40.899
Corydalis cava	-10.094	13.842	37.366
Corydalis solida	-18.657	5.05	29.134
Hepatica nobilis	-8.689	15.07	38.049

Table S7: Posterior estimated species-level random effects (means and 95% Bayesian credible intervals) for Eastern North American tree and wildflower species. Estimates are provided for the parameters derived from the JAGS model described in the main methods section.

	**Posterior estimates for JAGS model**		
Tree Species	2.5 BCI	Mean	97.5 BCI
Acer rubrum	-7.604	14.973	37.502
Acer saccharum	-6.973	15.713	38.429
Carya glabra	7.179	30.13	52.733
Fagus grandifolia	-4.443	18.569	41.082
Quercus alba	3.722	26.84	49.571
Quercus rubra	-1.691	20.884	43.75
Wildflower Species			
Anemone quinquefolia	5.866	28.665	51.847
Dicentra canadensis	0.89	23.615	46.774
Dicentra cucullaria	-3.67	19.123	42.092
Erythronium americanum	-5.344	17.539	40.383
Hepatica americana	-10.444	12.357	35.284
Sanguinaria canadensis	-11.023	11.935	34.784

Supplementary Figures

Figure S1: Maps of the 5,522 herbarium specimens collected between 1901 and 2020 across a) eastern Asia, b) western Europe, and c) eastern North America. Points represent observations of wildflower FFD (circles) and overstory LOD (crosses). Background color indicates average March-April temperatures in the current climate simulation (averaged from 2009-2018 using CRU TS v4.03 climate data).

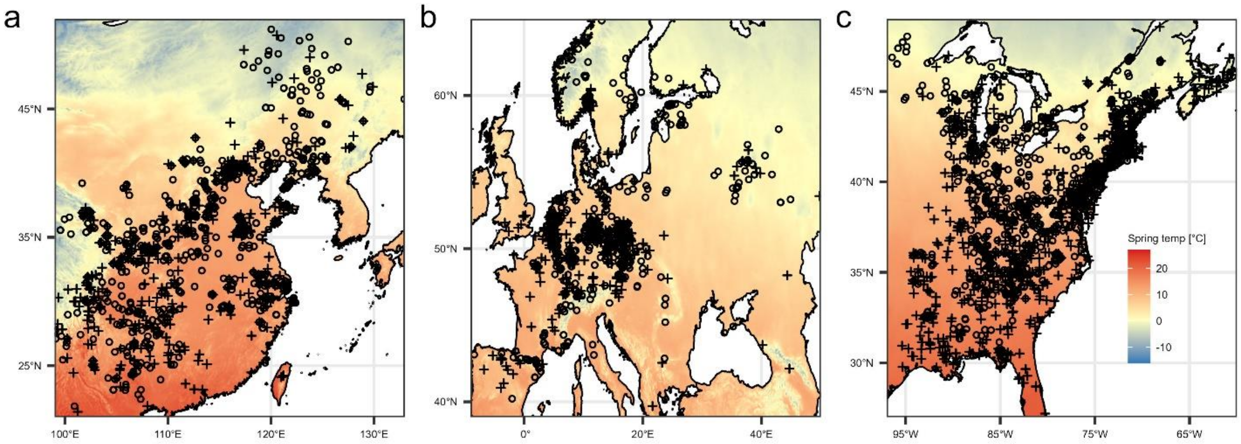

Figure S2: Posterior estimated means (left-hand panels) and standard deviations (right-hand panels) of spatial autocorrelation effects (in days) for (a-b) wildflower and (c-d) canopy tree phenology in East Asia. Positive and negative mean values indicate regions where phenology is expected to be delayed or earlier, respectively, compared to what is predicted from spring temperature and elevation alone. Maps are cropped to the extent of the Matérn meshes used to fit the models.

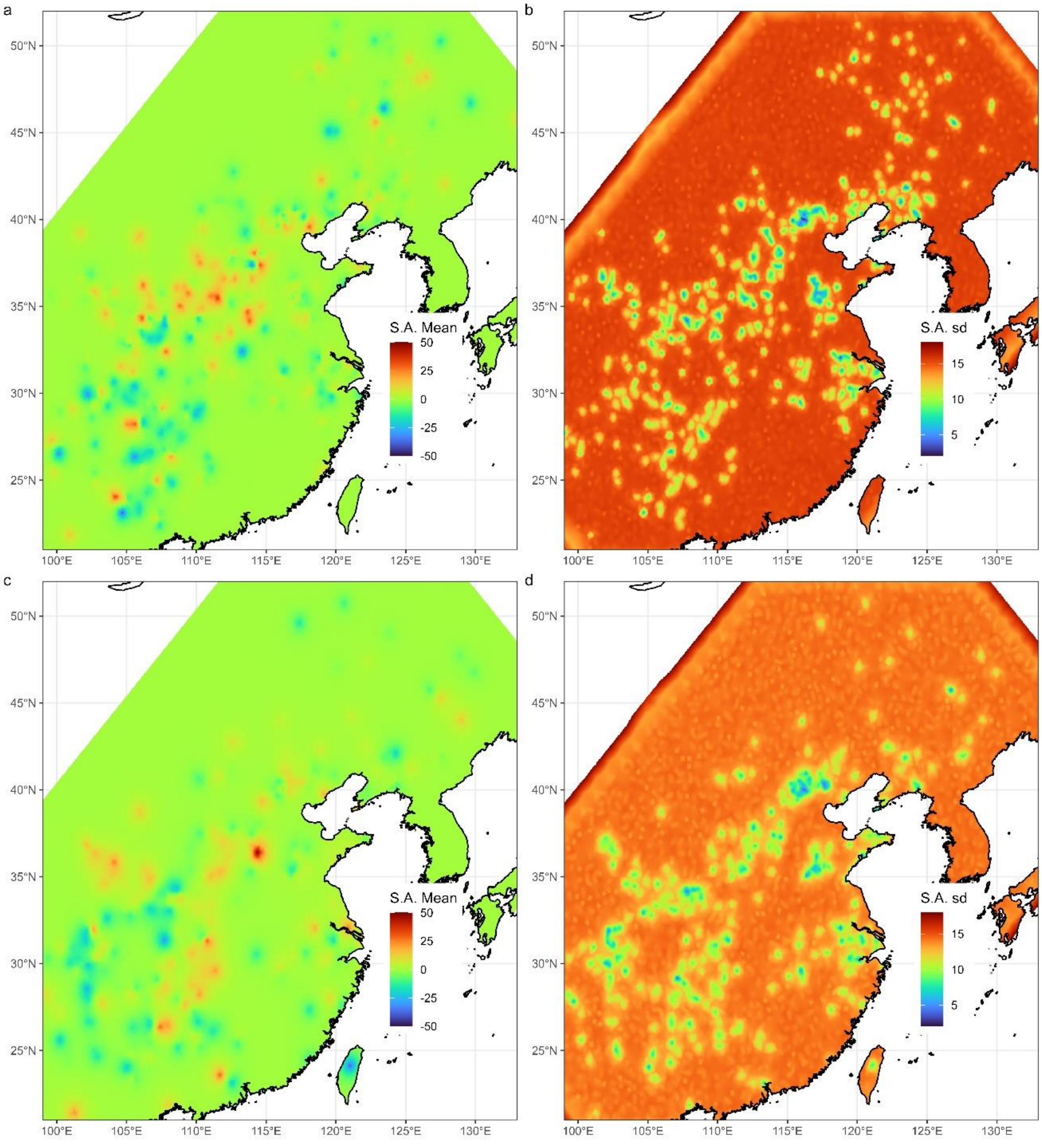

Figure S3: Posterior estimated means (left-hand panels) and standard deviations (right-hand panels) of spatial autocorrelation effects (in days) for (a-b) wildflower and (c-d) canopy tree phenology in Europe. Positive and negative mean values indicate regions where phenology is expected to be delayed or earlier, respectively, compared to what is predicted from spring temperature and elevation alone. Maps are cropped to the extent of the Matérn meshes used to fit the models.

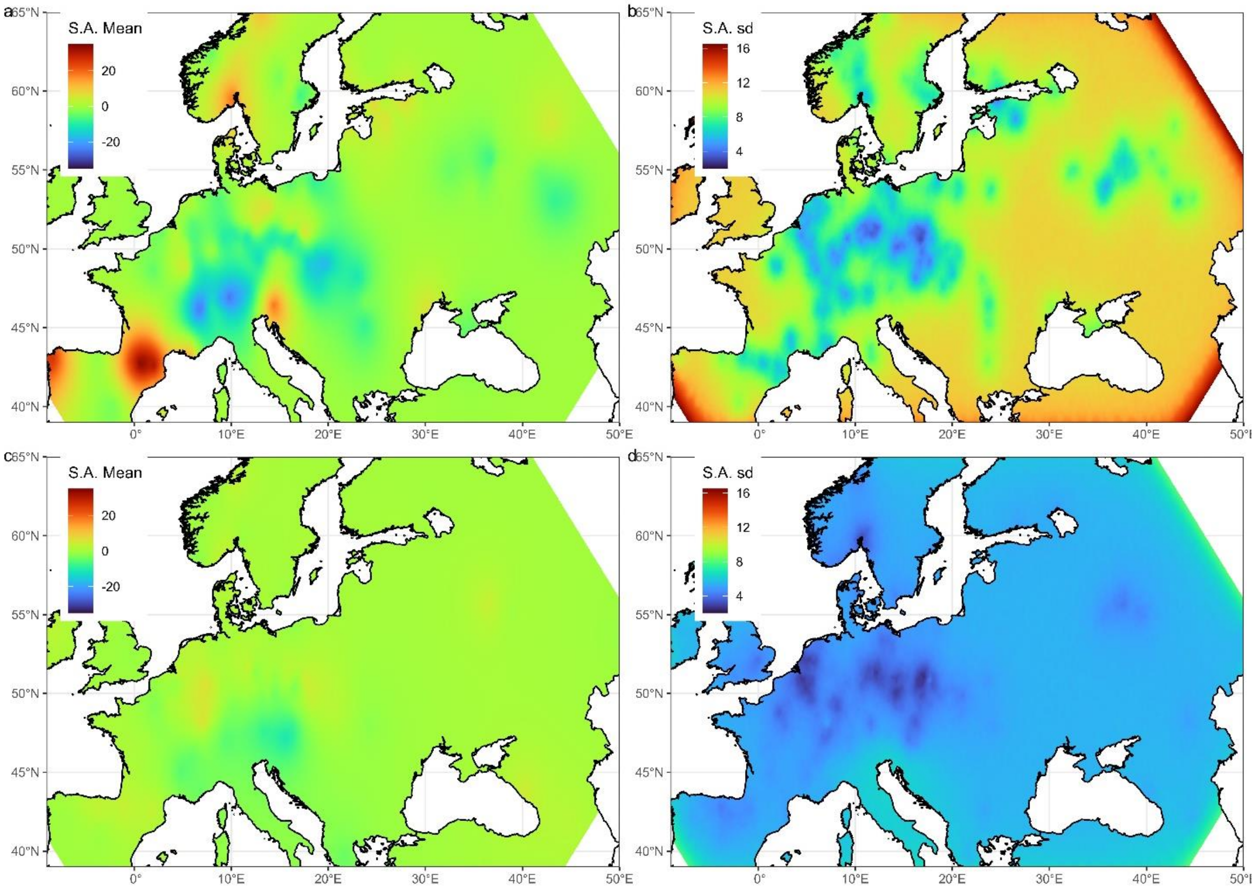

Figure S4: Posterior estimated means (left-hand panels) and standard deviations (right-hand panels) of spatial autocorrelation effects (in days) for (a-b) wildflower and (c-d) canopy tree phenology in eastern North America. Positive and negative mean values indicate regions where phenology is expected to be delayed or earlier, respectively, compared to what is predicted from spring temperature and elevation alone. Maps are cropped to the extent of the Matérn meshes used to fit the models.

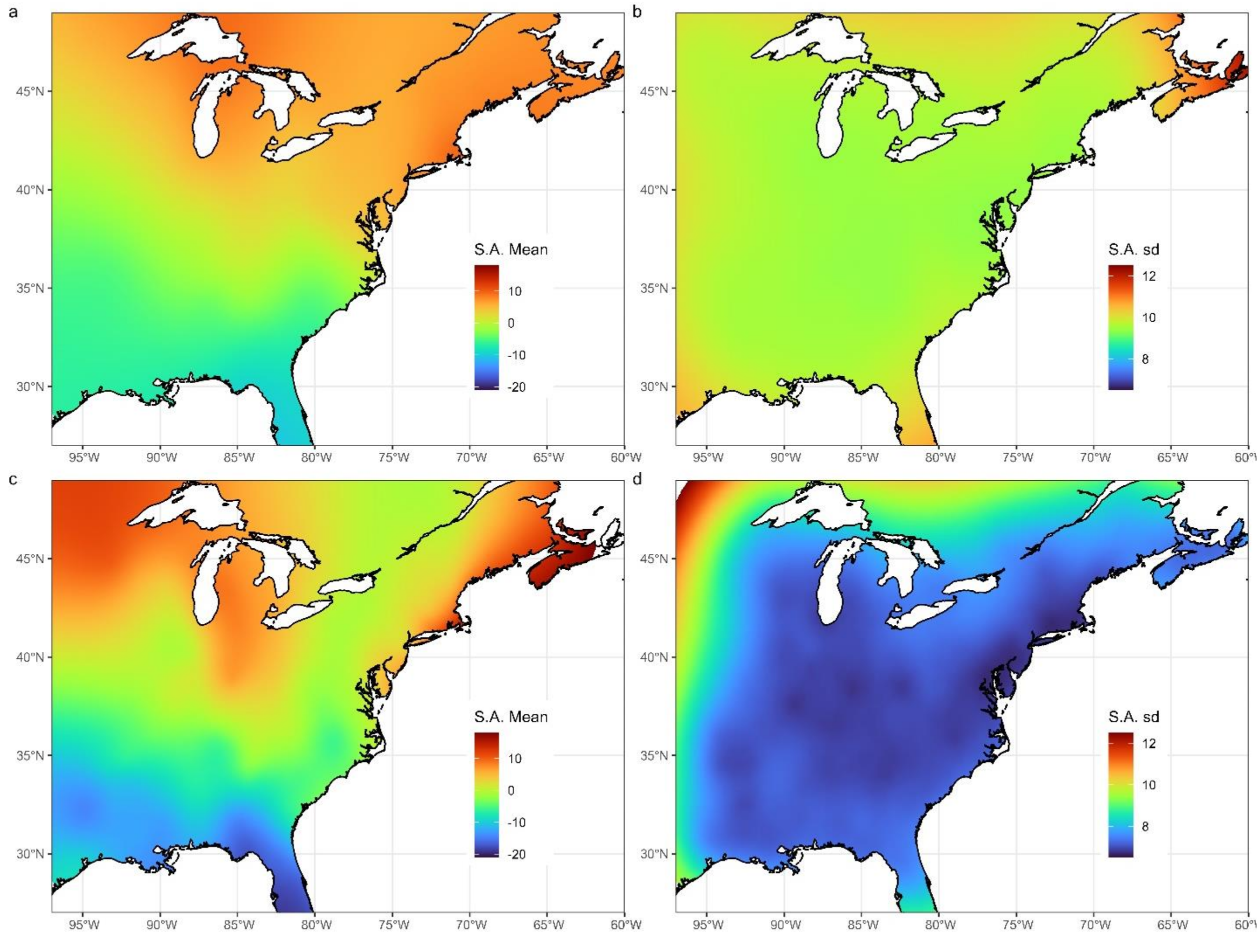

Figure S5: Estimated wildflower FFD (top row) and canopy tree LOD (bottom row) under current climate conditions (averaged from 2009-2018, see methods) in (a, d) Asia, (b, e) Europe, and c, f) North America.

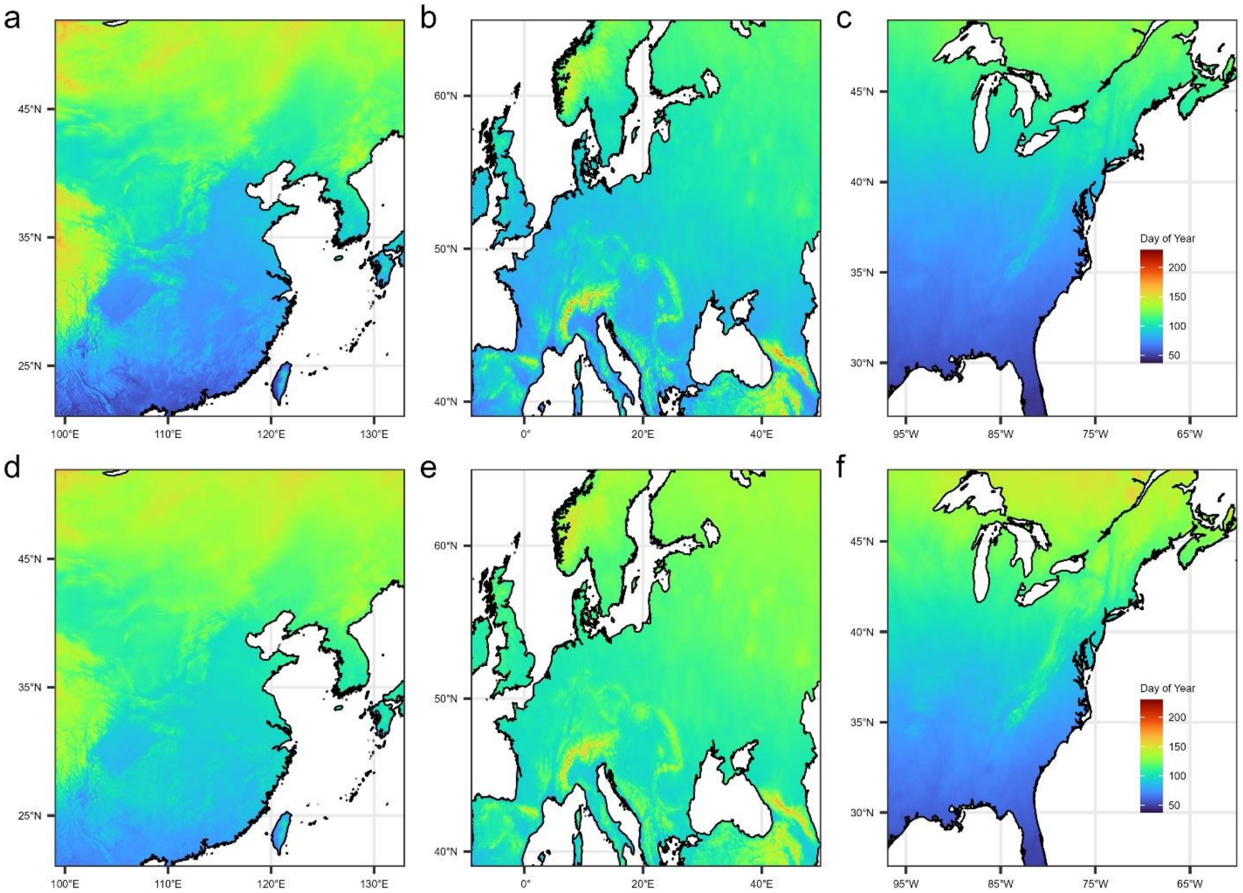

Figure S6: Projected wildflower FFD (top row) and canopy tree LOD (bottom row) under future climate conditions (estimated from the average projected climate conditions from 2081-2100, see methods) in (a, d) Asia, (b, e) Europe, and c, f) North America.

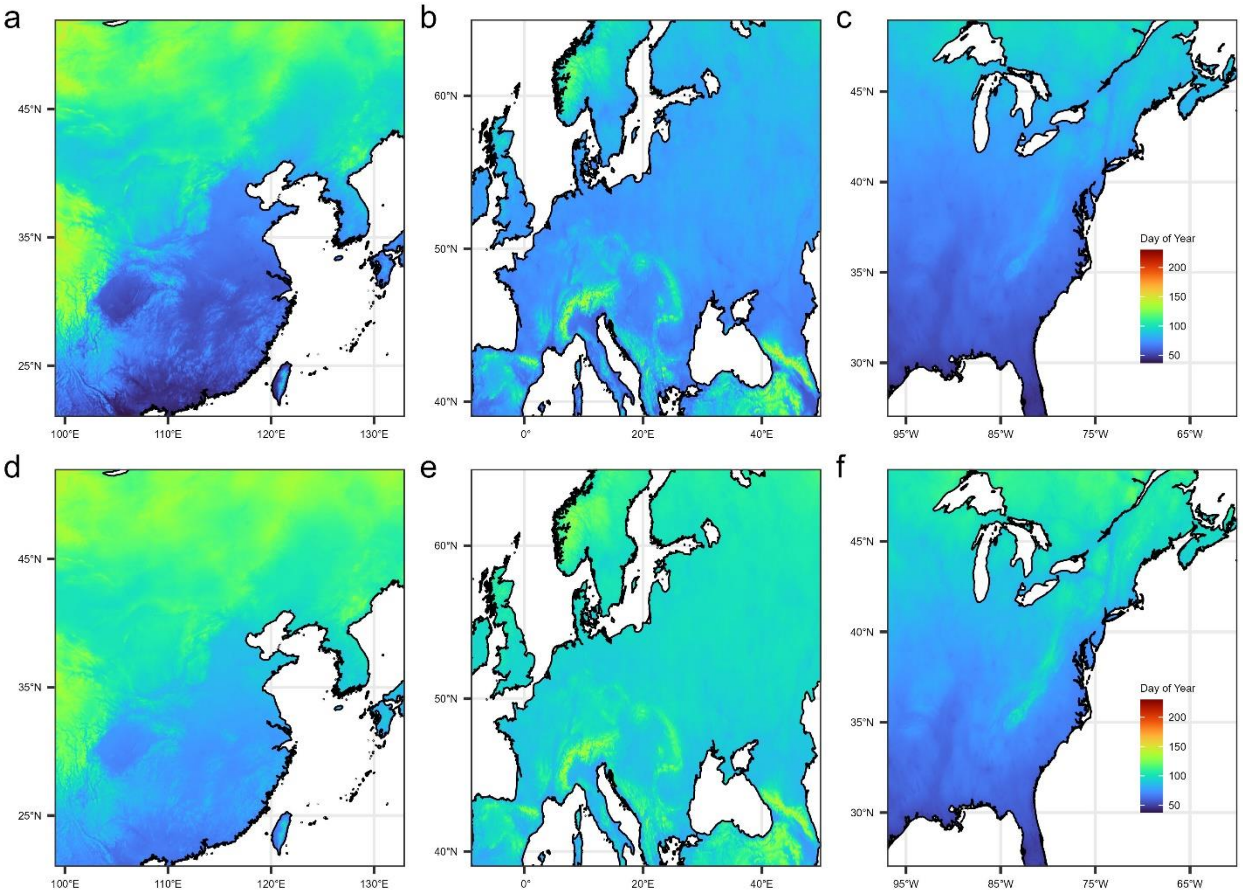

Figure S7: Projected mean difference between wildflower flowering (FFD) and canopy tree leaf out (LOD) (in days) under future climate conditions at the end of the century (see methods) in a) Asia, b) Europe, and c) North America. Negative values indicate LOD is estimated to occur before FFD.

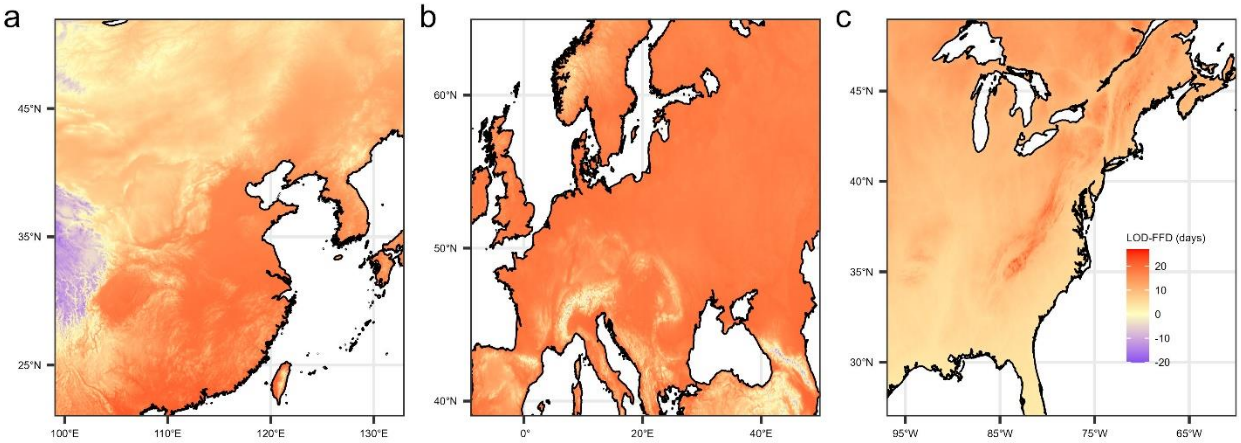

Figure S8: Difference in average spring temperature between current environmental conditions (averaged between 2009-2018) and future conditions (2080-2100) for a) Asia, b) Europe, and c) eastern North America. Dark gray regions indicate areas where the consensus land classification is < 1% deciduous or mixed deciduous forest cover.

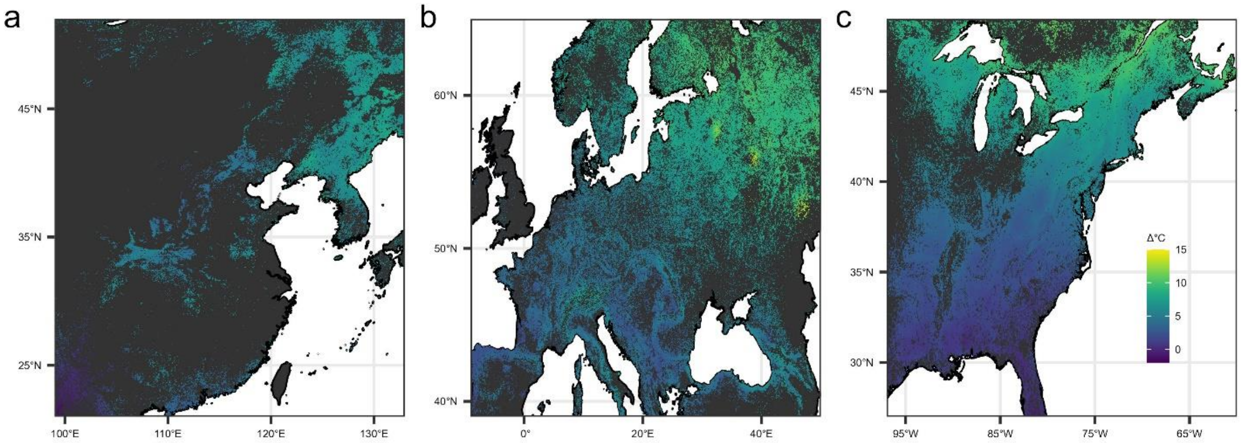

Figure S9: Correlation strengths (adjusted r^2 values) for linear regressions relating different one-, two-, and three-month aggregated spring temperature windows to observed wildflower First Flowering Date (FFD, blue) and tree Leaf Out Date (LOD, gold). Horizontal lines indicate the adjusted r^2 value for the relationship with average March-April temperature for ease of comparison with the other windows.

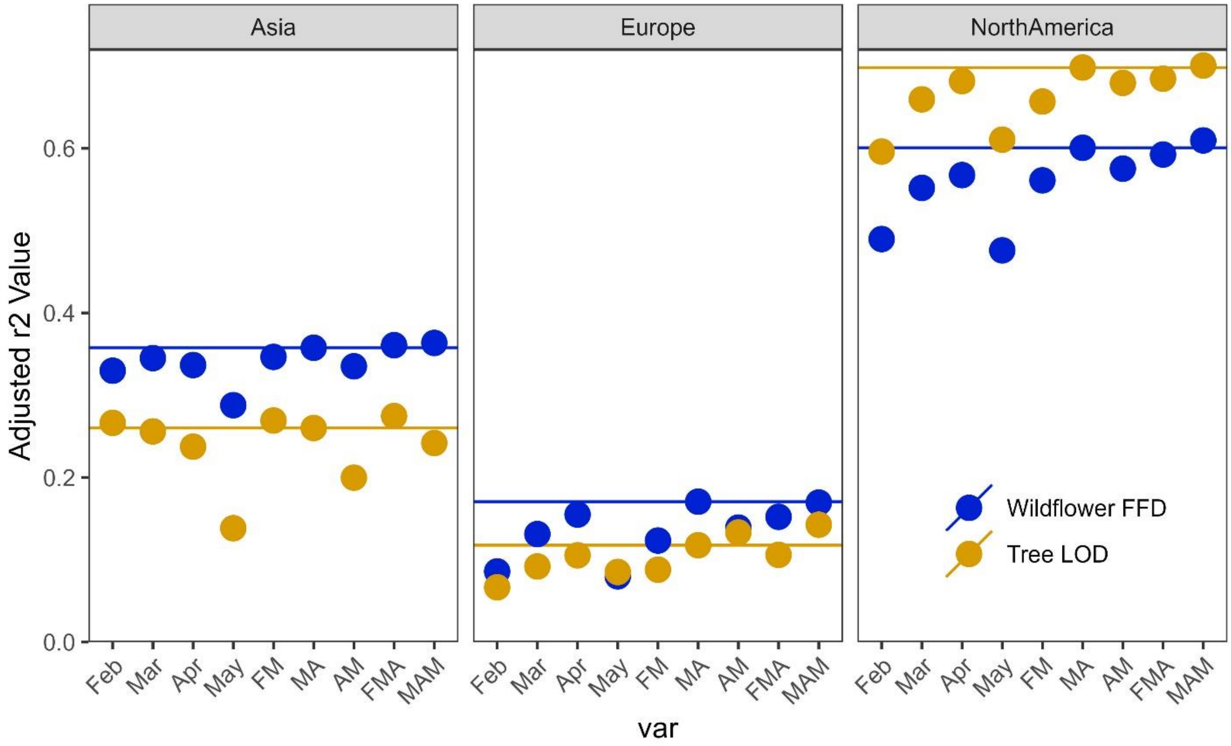

Figure S10: Matérn meshes used to fit INLA models that include spatial autocorrelation terms for a) East Asia, b) Europe, and c) Eastern North America. Red and blue points represent relative locations of observed wildflower and tree phenology, respectively. Coordinates are scaled from decimal degree coordinates so that all locations have positive latitude and longitude values.

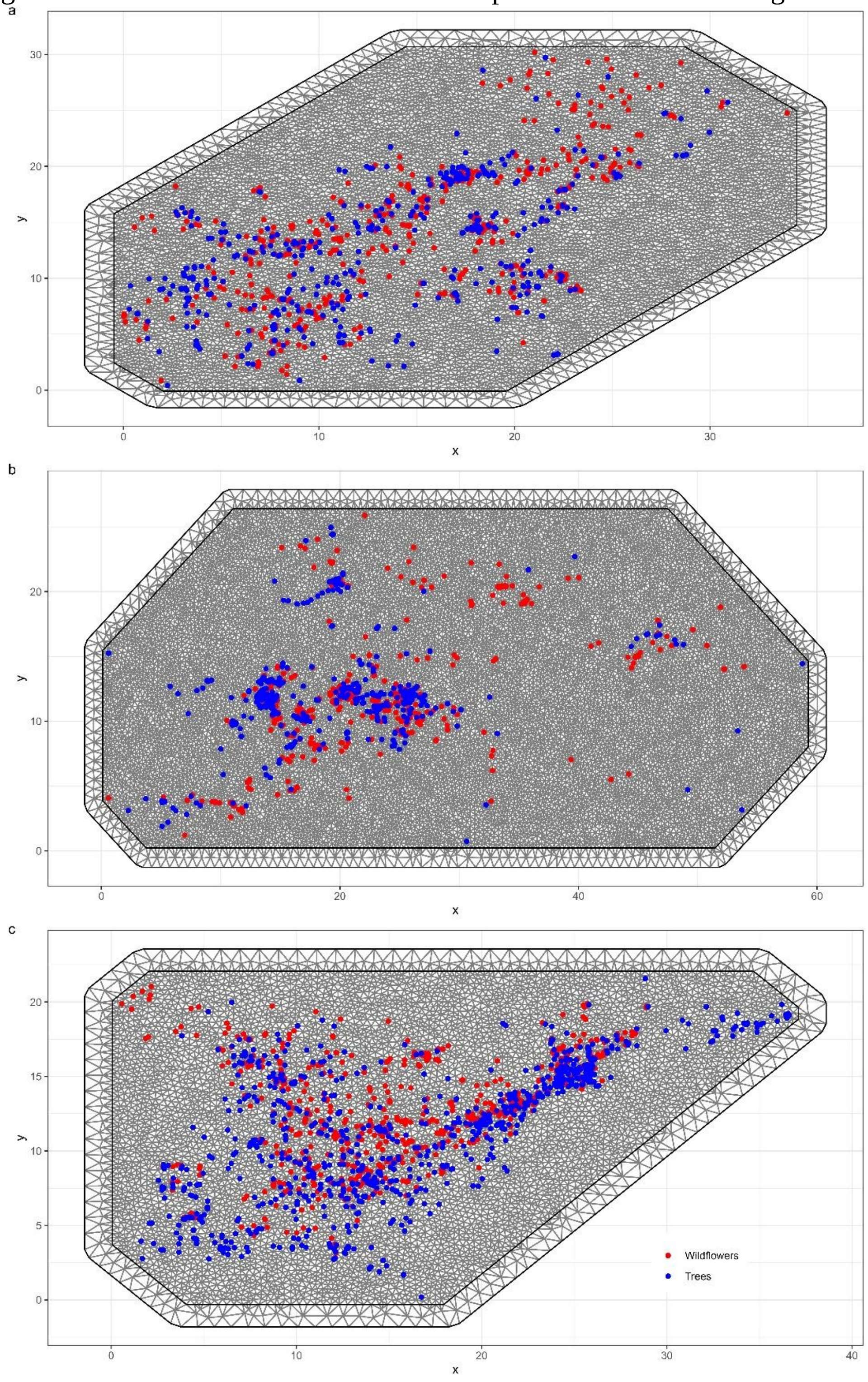

Figure S11: Full, uncropped version of Figure 3.

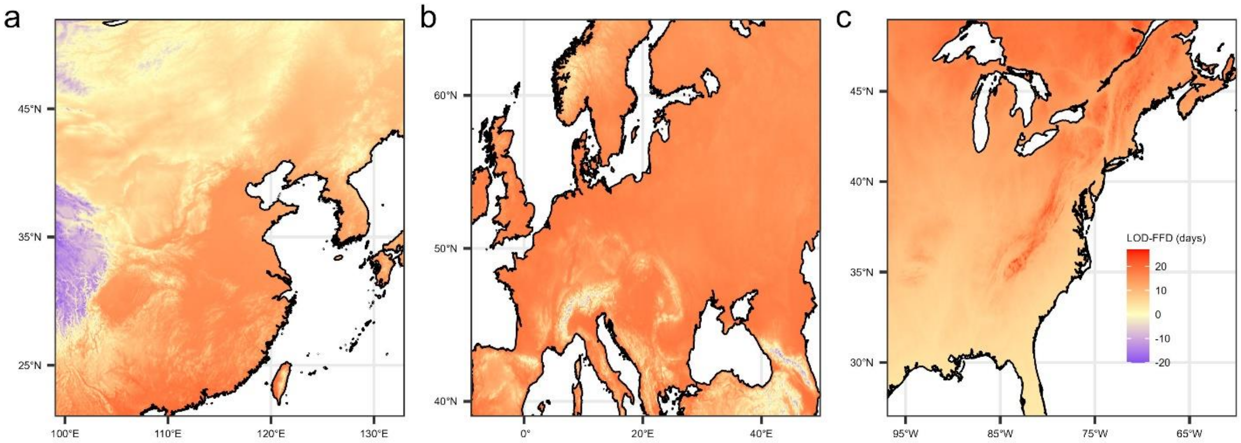

Figure S12: Full, uncropped version of Figure 4.

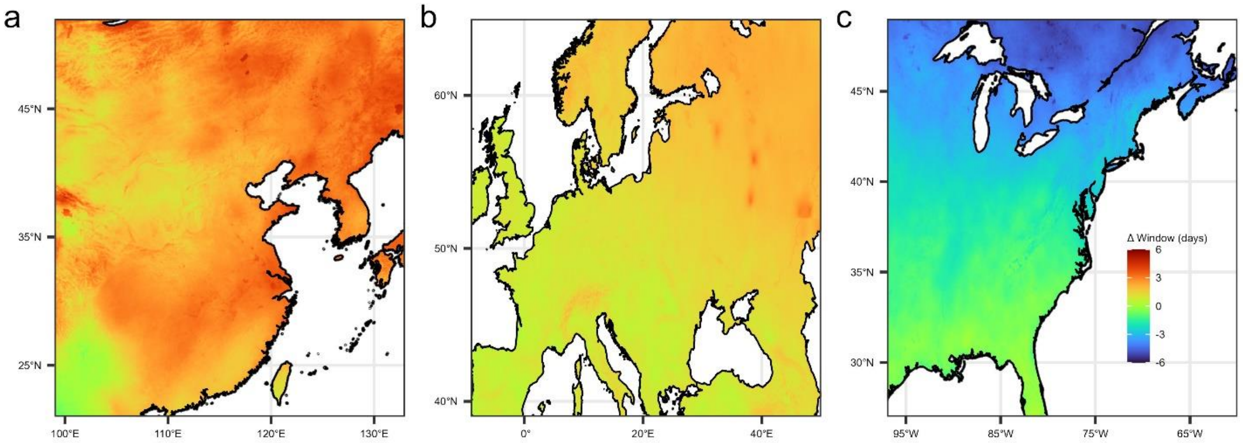

Supplementary Information References:

1. Ettinger, A. K. *et al.* Winter temperatures predominate in spring phenological responses to warming. *Nature Climate Change* **10**, 1137–1142 (2020).
2. Willems, F. M., Scheepens, J. F. & Bossdorf, O. Forest wildflowers bloom earlier as Europe warms: lessons from herbaria and spatial modelling. *New Phytologist* **235**, 52–65 (2022).
