# Supplementary material for: Wildflower phenological escape differs by continent and spring temperature
Source: Nat Commun. 2022 Nov 22;13:7157. doi: 10.1038/s41467-022-34936-9 (PMC9684453; doi:10.1038/s41467-022-34936-9)
Supplement: Supplementary file 2 — Reporting Summary [file 41467_2022_34936_MOESM2_ESM.pdf]

nature portfolio

Corresponding author(s): Benjamin R Lee

Last updated by author(s): Nov 8, 2022

Reporting Summary

Nature Portfolio wishes to improve the reproducibility of the work that we publish. This form provides structure for consistency and transparency in reporting. For further information on Nature Portfolio policies, see our Editorial Policies and the Editorial Policy Checklist.

Statistics

For all statistical analyses, confirm that the following items are present in the figure legend, table legend, main text, or Methods section.

n/a	Confirmed	
[ ]	[x]	The exact sample size (n) for each experimental group/condition, given as a discrete number and unit of measurement
[ ]	[x]	A statement on whether measurements were taken from distinct samples or whether the same sample was measured repeatedly
[x]	[ ]	The statistical test(s) used AND whether they are one- or two-sided<br>*Only common tests should be described solely by name; describe more complex techniques in the Methods section.*
[ ]	[x]	A description of all covariates tested
[ ]	[x]	A description of any assumptions or corrections, such as tests of normality and adjustment for multiple comparisons
[ ]	[x]	A full description of the statistical parameters including central tendency (e.g. means) or other basic estimates (e.g. regression coefficient) AND variation (e.g. standard deviation) or associated estimates of uncertainty (e.g. confidence intervals)
[x]	[ ]	For null hypothesis testing, the test statistic (e.g. F, t, r) with confidence intervals, effect sizes, degrees of freedom and P value noted<br>*Give P values as exact values whenever suitable.*
[ ]	[x]	For Bayesian analysis, information on the choice of priors and Markov chain Monte Carlo settings
[ ]	[x]	For hierarchical and complex designs, identification of the appropriate level for tests and full reporting of outcomes
[x]	[ ]	Estimates of effect sizes (e.g. Cohen's d, Pearson's r), indicating how they were calculated

Our web collection on statistics for biologists contains articles on many of the points above.

Software and code

Policy information about availability of computer code

Data collection	Climate data, both historical and future climate projections, were extracted from the Climate Research Unit gridded Temperature Series (CRU TS) data set v4.05 using the georeferenced location for each specimen. This extraction was conducted in R V 4.1.0
Data analysis	Bayesian model analysis was conducted in R primarily using the "r2jags" (for the models presented in the main manuscript) and "inlabru" (for those described in the supplement) packages. All maps and figures presented in this manuscript were constructed in R V4.1.0, primarily using the "stars", "sp", and "ncdf4" packages. Example code is provided in a freely accessible online depository as noted in the text.

For manuscripts utilizing custom algorithms or software that are central to the research but not yet described in published literature, software must be made available to editors and reviewers. We strongly encourage code deposition in a community repository (e.g. GitHub). See the Nature Portfolio guidelines for submitting code & software for further information.

Data

Policy information about availability of data

All manuscripts must include a data availability statement. This statement should provide the following information, where applicable:

- Accession codes, unique identifiers, or web links for publicly available datasets
- A description of any restrictions on data availability
- For clinical datasets or third party data, please ensure that the statement adheres to our policy

The individual specimen phenology and environmental conditions data generated in this study have been deposited in the Zenodo database at https://www.doi.org/10.5281/zenodo.7080193. The processed spatial autocorrelation data are available in the same location. Past, current, and future climate estimates

were acquired from the WorldClim 2.1 [https://www.worldclim.org/data/index.html] and CRU TS4.03 [https://crudata.uea.ac.uk/cru/data/hrg/] datasets.

Human research participants

Policy information about studies involving human research participants and Sex and Gender in Research.

Reporting on sex and gender	Not applicable
Population characteristics	Not applicable
Recruitment	Not applicable
Ethics oversight	Not applicable

Note that full information on the approval of the study protocol must also be provided in the manuscript.

Field-specific reporting

Please select the one below that is the best fit for your research. If you are not sure, read the appropriate sections before making your selection.

☐ Life sciences ☐ Behavioural & social sciences ☒ Ecological, evolutionary & environmental sciences

For a reference copy of the document with all sections, see nature.com/documents/nr-reporting-summary-flat.pdf

Ecological, evolutionary & environmental sciences study design

All studies must disclose on these points even when the disclosure is negative.

Study description	We investigated differences in phenological sensitivity between understory wildflowers and canopy tree species across three continents using hierarchical Bayesian models. Models were fit using information from 5,522 herbarium specimens. The models presented in this manuscript were selected from a model selection process and included fixed effects of spring temperature and elevation and species-level random effects. Models were run separately by continent and stratum (tree vs. wildflower).
Research sample	The research sample included 5,522 herbarium specimens from across eastern North America, Europe, and eastern Asia. We chose six native wildflower species and six native canopy tree species from each continent (with the exception of China, which included 10 native tree species). Our rationale for species choice was all species are deciduous and all wildflower species are spring ephemerals, which was used as a control to ensure that flowering of these species was a good approximation of spring emergence. Further, we species were chosen based on greatest data availability from the years 1901-2020, when climate approximations were available.
Sampling strategy	Herbarium specimens were selected first by species (as described above). We downloaded digitized images of all individuals of each species from within its native continent. Specimens were then removed if they were missing location data needed for geolocation, if they did not exhibit the desired phenophase (i.e., if wildflowers were not yet or were past flowering), or if they were collected before 1901. We used all data that met this criterion.<br><br>No sample size calculation was performed during this analysis. We used the data available to us, limited in extent by historical collection availability. We considered this to be sufficient given the wide temporal and geographic range exemplified in our dataset.
Data collection	Data collection was led by TK Miller, C Rosche, and Y Yang for herbarium data originating from North America, Europe, and Asia, respectively. Data were recorded either using pen and paper or were directly electronically entered into spreadsheets.
Timing and spatial scale	Herbarium collections were identified and specimen-level data were extracted between January and April 2021. Specimens on each continent were accessed at a single data per continent. Specimens were also scored and georeferenced in 2021, but herbarium specimens ranged in date from 1901-2020. Earlier specimens were available, but we chose not to include them because we were only able to access historical climate approximations dating to 1901. We accessed all available (i.e., fully digitized) specimens from each of the chosen species (see Sampling strategy). The specimens were collected across three continents (North America, Asia, and Europe), spanning latitudes 21.4-64.9 degrees and longitudes -96.4 to 132.9 degrees.
Data exclusions	Specimens were then removed if they were missing location data needed for geolocation, if they did not exhibit the desired phenophase (i.e., if wildflowers were not yet or were past flowering), or if they were collected before 1901. This criteria were preestablished and are consistent with previously published and validated methods.
Reproducibility	Data reproducibility is accounted for by thorough documentation of the herbarium specimens used in the analysis. These data sets will also be published upon acceptance for publication. Analysis reproducibility is accounted for by publication of R code used to fit the models upon acceptance for publication.
Randomization	Observations were grouped by species and models include species-level random effects to account for possible differences in phenological sensitivity.
Blinding	Blinding was not relevant to our study as the identifying information of each specimen (e.g., species) was integral to the study and

necessary for analysis.

Did the study involve field work? ☐ Yes ☒ No

Reporting for specific materials, systems and methods

We require information from authors about some types of materials, experimental systems and methods used in many studies. Here, indicate whether each material, system or method listed is relevant to your study. If you are not sure if a list item applies to your research, read the appropriate section before selecting a response.

Materials & experimental systems

n/a	Involved in the study
☒	☐ Antibodies
☒	☐ Eukaryotic cell lines
☒	☐ Palaeontology and archaeology
☐	☒ Animals and other organisms
☒	☐ Clinical data
☒	☐ Dual use research of concern

Methods

n/a	Involved in the study
☒	☐ ChIP-seq
☒	☐ Flow cytometry
☒	☐ MRI-based neuroimaging

Animals and other research organisms

Policy information about studies involving animals; ARRIVE guidelines recommended for reporting animal research, and Sex and Gender in Research

Laboratory animals	This study did not involve lab animals
Wild animals	This study did not involve wild animals
Reporting on sex	Sex was not relevant in this research
Field-collected samples	All specimens were collected in the field, pressed, and mounted to herbarium sheets. Specimens were then digitized and accessed online.
Ethics oversight	No ethical approval or guidance was required for this study. We studied only plants and our sampling was non-destructive.

Note that full information on the approval of the study protocol must also be provided in the manuscript.
